# A study on the properties and working mechanism of a waterborne polyurethane-modified silicate-based coating

**DOI:** 10.1039/c9ra04441h

**Published:** 2019-08-27

**Authors:** Hui Yuan, Yushuai Wang, Zhiyong Liu, Shiyu Li

**Affiliations:** College of Civil Engineering, Yantai University Yantai 264005 China

## Abstract

Herein, the effects of the amount of waterborne polyurethane, silica sol and fillers on the compressive and bending strength, temperature resistance and acid resistance of waterborne polyurethane-modified silicate-based coatings were investigated. The results indicated that the modified coating showed higher mechanical properties, impermeability and bonding properties when the amounts of polyurethane and silica sol were 10% and 4%, respectively. The room temperature strength, temperature resistance and acid resistance of the modified coating were 25.1%, 34.1% and 32.4% higher than those of unmodified coatings, respectively. Moreover, the flexibility of the coating was significantly improved. The compression–bend ratio of the modified coating was 7% higher than that of the unmodified coating. The impermeability of the modified coating was 53% higher than that of the unmodified coating. The bond strengths of the modified coatings with a concrete and an acid-resistant ceramic tile were 3.08 MPa and 5.84 MPa, respectively, which were higher than the standard value of 1.2 MPa. SEM analysis showed that the morphological structure of the coating was changed. The results showed that a dense micro-structure with an interpenetrating network was formed. EDS analysis showed that the sulfur atom was absent in the modified coating after acid storage. The MIP test showed that the porosity of the modified sample decreased and the pore distribution was improved. TGA analysis showed that the modified coating could meet the requirement of temperature resistance at 250 °C.

## Abstract 2

Herein, the effects of the amount of waterborne polyurethane, silica sol and fillers on the compressive and bending strength, temperature resistance and acid resistance of waterborne polyurethane-modified silicate-based coatings were investigated.

## Graphical abstract

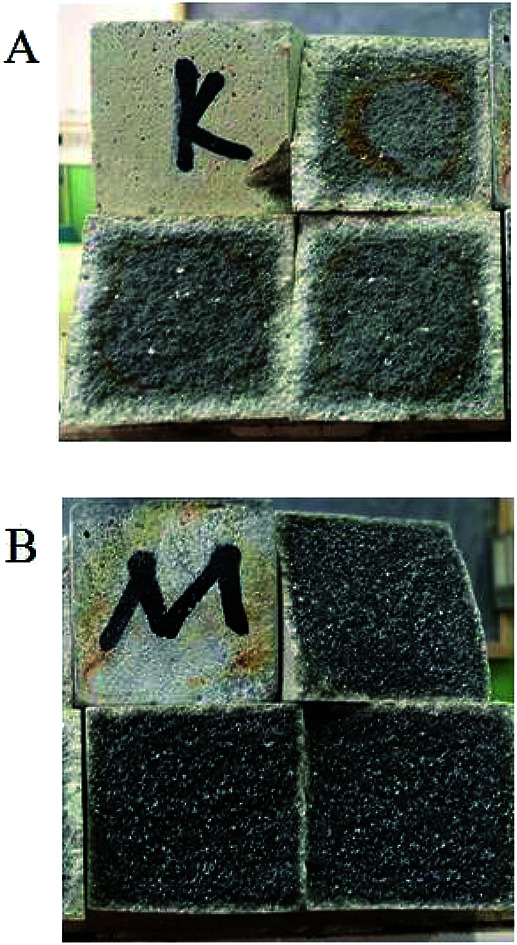

## 1. Introduction

Anti-corrosive coatings have been widely used in the construction, petrochemical, transportation, electric power and other industries.^1–5^ The flue gas produced by industrial boilers contains a large amount of sulfur dioxide, which has strong corrosiveness to chimneys and will seriously affect the durability of buildings.^6–10^ Therefore, effective desulfurization should be carried out before the discharge of flue gases. However, the flue gas treated by the flue gas desulfurization system was still corrosive to chimneys.^11,12^ Higher requirements were then put forward for the performance of anti-corrosive coatings. Therefore, the preparation of high-temperature resistant and anti-corrosive coatings as chimney linings to effectively protect the chimney from damages caused by corrosive materials is urgently needed.

The anti-corrosion development of flue chimneys ranged from the development of OM coatings, high-temperature resistant coatings and KP1 cement, to glass fiber-reinforced plastics (FRP), glass flake cement and cement-bonded lining with vinyl ester resin as the main base material, to a vinyl ester resin flake cement FRP composite, and a vinyl cement-bonded FRP-insulated industrial ceramic brick lining.^13^ At present, the main anti-corrosion solutions for chimney lining include the use of special acid-resistant cement, glass flake cement, foamed acid-resistant glass bricks and anti-corrosion coatings.^14^ Traditional inorganic high-temperature resistant coatings have excellent temperature resistance; however, their applications have been limited to a certain extent due to the brittleness of film-forming materials, poor bonding performance, insufficient water resistance and strict requirements for the treatment of substrates.^15,16^ Therefore, many researchers started to utilize the special properties of new materials to modify sodium silicate by adding inorganic acid and organic salt-modified adhesives; however, the results showed that the pH value was changed dramatically, which in turn destroyed the structure of the adhesives, and thus, the modification effect was poor.^17^ The effect of modifying the adhesives was not obvious. However, organic–inorganic composites with excellent properties could be obtained by mixing organic and inorganic materials.^18–23^ Koester anti-corrosive coatings in Germany are a kind of special mortar with strong acid resistance based on polymers and silicate, which have high compressive strength, flexibility, acid resistance and bending resistance. The purpose of the addition of organic components was to improve the physical and mechanical properties, such as adhesion, impact resistance and flexibility, of the coatings. A polymer was used to modify the sodium silicate acid-resistant mortar. The results showed that the porosity of the acid-resistant mortar was decreased, and the compactness was improved by the addition of polyvinyl acetate and vinyl acetate latex.^24^ The modified epoxy resin was added to a water glass acid-resistant anti-corrosive material to form a three-dimensional network structure *via* cross-linking by a curing agent, which improved the impermeability, mechanical strength and corrosion resistance. Polyurethane possesses the properties of light weight, low viscosity, good permeability, low thermal conductivity and high mechanical performance, and the water glass has high thermal stability, low cost and a non-toxic nature.^16,25–28^ Therefore, the preparation of inorganic–organic composite high-temperature corrosion-resistant coatings can significantly solve the corrosion problem of chimneys. The properties and the mechanism of the waterborne polyurethane-modified silicate-based coating were studied. The effects of the amount of waterborne polyurethane, silica sol and fillers on the compressive and bending strength, temperature resistance and acid resistance of the coating were investigated. Moreover, the micro-structure of the coating was analyzed.

## 2. Experiments

### 2.1. Materials

Commercial water glass (Na_2_O·*n*SiO_2_·*m*H_2_O, modulus 2.8) was purchased from Haixing Yongshun Water Glass Manufacturing Co., Ltd. (Cangzhou, China). Commercial waterborne polyurethane (solid content about 32%, not less than 30%) was purchased from Qingdao Jubang New Materials Co., Ltd. (Qingdao, China). Commercial quartz powder and quartz sand (1200 mesh and 80 mesh) were purchased from Quancheng Quartz Sand Factory (Yantai, China). Commercial cast stone powder (120 mesh) was purchased from Baoding Casting Stone Group Co., Ltd. (Baoding, China). Chemical silica sol (30%) was obtained from Jinghuo Technology Glass Co., Ltd. (Dezhou, China). Chemical sulfuric acid (40%) was purchased from Yantai Sanhe Chemical Reagent Co., Ltd. (Yantai, China).

### 2.2. Preparation

Herein, a waterborne polyurethane-modified silicate-based coating was prepared by a mechanical mixing method. The planetary cement mortar mixer (model JJ-5) was purchased from Ningbo Taifu Industry and Trade Co., Ltd. (Ningbo, China). Water glass, silica sol and waterborne polyurethane with the weight ratio of 86 : 4 : 10 were added to the mixer and stirred for 5 minutes at 120 rpm, and the resulting material was denoted as component A; quartz sand, quartz powder and cast stone powder with the weight ratio of 30 : 28 : 42 were added to another mixer and stirred for 5 minutes at 120 rpm, and the resulting material was denoted as component B. The abovementioned two components were mixed for 5 minutes at 120 rpm, and then, the coating was achieved at room temperature.

### 2.3. Experimental methods

#### 2.3.1 Mechanical property tests

The compressive strength and bending strength were taken as the test indices of mechanical properties. All the compressive strength and bending strength measurements were carried out using a universal testing machine (Model WDW-100) and a bending testing machine (Model DKZ-5000) at room temperature, respectively. Herein, six specimens of 40 mm × 40 mm × 160 mm size were prepared according to DL/T 901-2004 “Acid-resisting materials of chimney lining for fossil fuel power plant”.^29^ Temperature resistance was reflected by the residual rate of the mechanical properties of the specimens after being kept at 250 °C for 4 h. The mechanical properties of the specimens that were immersed in 40% sulfuric acid for 30 days reflected their acid resistance. The specimens were naturally cured for 15 days in an air environment at 23 ± 2 °C and the relative humidity of less than 80% and strictly prohibited from being in contact with water or steam during maintenance.

#### 2.3.2. Impermeability test

The impermeability test was carried out according to GB 50212-2014 “Code for the construction of building anti-corrosion engineering”.^30^ Stirred coatings were put in the impermeability test mold coated with organic oil. The size of the test mold was 80 mm at the bottom, 70 mm at the top and 30 mm at the top. The test mold was placed on the shaking table and vibrated until the surface was pulped, and the bubbles were eliminated. The test mold was smoothed with a spatula, 3 pieces per group. The cured specimens were coated with melted paraffin. The top and bottom of the specimens were not coated. The specimens were loaded onto the impermeable mold with a certain degree of heat. Finally, the specimens were installed on the impermeability test machine.

#### 2.3.3. Bond strength test

The bond strength test was carried out according to GB 50212-2014 “Code for the Construction of Anti-Corrosion Engineering of Buildings” A 3.6.9.^30^ The concrete blocks with the size of 100 mm × 100 mm × 50 mm were used as the test base, and the surface of the concrete blocks after curing was slightly polished. The coatings were uniformly coated on the surface of concrete blocks. The coatings were cured in a 2.3.1 way. After curing to aging, the specimens were opened with an opener, and the coating should be cut through to the concrete base. Then, the tensile test column was fixed to the cut coating with a special structural adhesive.

#### 2.3.4. SEM (EDS), MIP and TGA tests

SEM (EDS) was performed using the Hitachi S-4800 field-emission scanning electron microscope (Hitachi Ltd., Tokyo, Japan). The resolution of the scanning electron microscope was not more than 1.0 nm, and the acceleration voltage was 0.5–30 kV. By increasing the resolution by 500, 2000, 10 000 and 100 000 times, the micro-morphology of the cross-section in different environments was observed. The EDS test was used to analyze the corrosive conditions of the coating by detecting corrosive medium elements and contents. The pore structure of the coating was investigated by mercury intrusion porosity (MIP). The pore volume was obtained by measuring the amount of mercury entering the pore under different external pressures. The thermogravimetric analysis (TGA) was conducted between room temperature and 1100 °C using the STA449F5 thermal analyzer system at the heating rate of 10 °C min^−1^ under a N_2_ atmosphere. The samples were taken at a distance of 10 mm from the surface of the specimens for the TGA of the unmodified group. The thermogravimetric analysis was carried out for the modified group at the depths of 10 mm and 20 mm from the surface of the specimens.

## 3. Results and discussion

### 3.1. Mechanical performance test results

The test results of the mechanical properties of the coatings are presented in Table 1. The average bending strength of the modified coatings at room temperature reached 10.00 MPa, which was 57% higher than that of the unmodified coating (6.38 MPa). The average compressive strength of the modified coatings reached 27.90 MPa, which was 25.1% higher than that of the unmodified coatings (22.3 MPa). The obtained compressive strength was significantly higher than the recommended value in the code. After the samples were subjected to 250 °C temperature for 4 h, the bending and compressive strengths of the modified group and the unmodified group were improved. The reason was that high temperature was conducive to the complete removal of water from sodium silicate and promoted the formation of silica crystals in sodium silicate. Therefore, the strengths of the coating were improved. Moreover, there was no abnormality, such as cracks, swelling, peeling and deformation of the coating, after the modified coating was subjected to high temperatures; the temperature resistance of the modified coating could fully meet the application requirements.

**Table 1:** Mechanical properties of the coatings^a^

Group	Bending strength (MPa)	Ratio	Compressive strength (MPa)	Ratio
A1	6.38	—	22.30	—
A2	10.00	—	27.90	—
B1	6.48	1.02	27.10	1.22
B2	10.05	1.01	36.35	1.30
C1	6.03	0.95	20.55	0.92
C2	10.04	1.00	27.21	0.96

a A1, blank group at room temperature; A2, modified group at room temperature; B1, blank group after suffering high temperature; B2, modified group after suffering high temperature; C1, blank group after acid storage; and C2, modified group after acid storage.

**Fig. 1:**
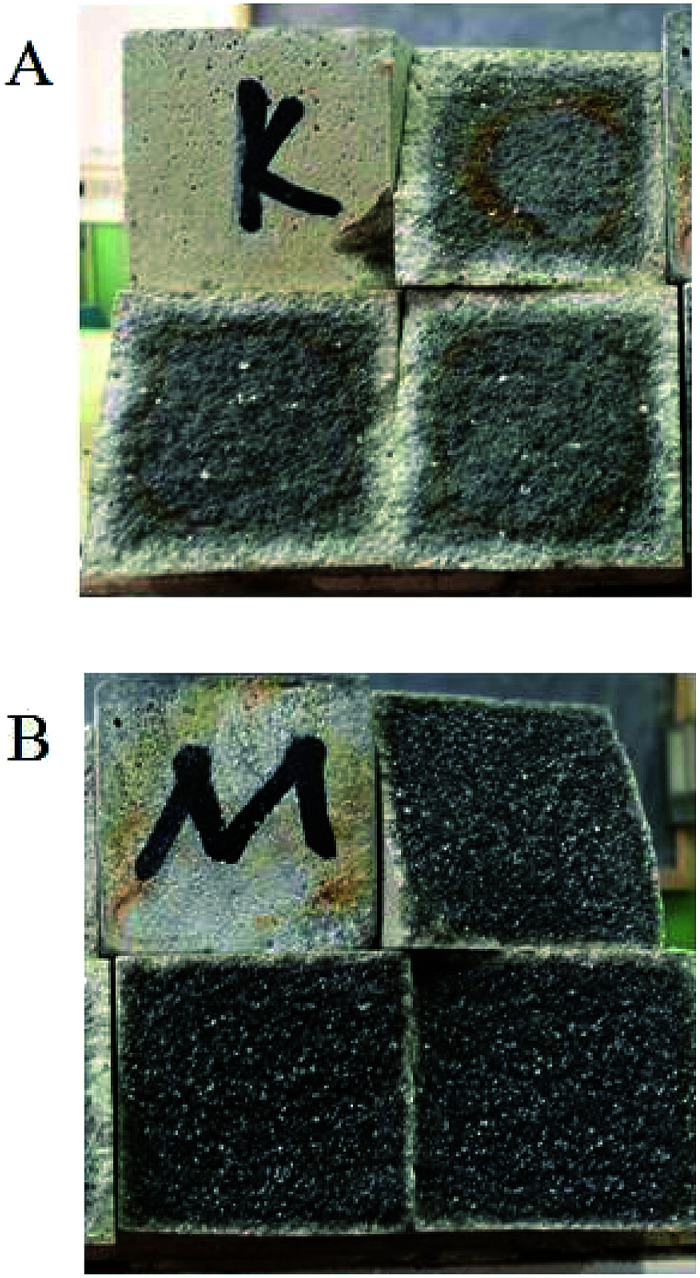
Sectional view of the coatings after acid storage: (A) blank group and (B) modified group.

In Table 1, it is also shown that after the samples are stored in 40% sulfuric acid for 30 days, the strength of the modified group decreases slightly and that of the blank group decreases significantly. The average compressive strength of the modified group after acid storage was 27.21 MPa, which was 32.4% higher than that of the blank group (20.55 MPa). Therefore, the modified coating has excellent acid resistance, and the strength change value is 11% higher than the standard value of 0.9. The appearances of the coating specimens after acid storage are shown in Fig. 1. It was clearly observed that the acid corrosion layer of the modified coating was relatively thin, which indicates that the modified coating has excellent acid resistance. However, the corrosive medium had penetrated the blank coating to a great extent. Moreover, the modified coating did not show any signs of corrosion, peeling, cracks, expansion and partial bubbling. Therefore, the acid resistance performance of the modified coating was stable.

### 3.2. Impermeability and bond strength test results

Table 2 shows the impermeability test results of the coating. As observed from Table 2, the impermeability of the coatings modified with waterborne polyurethane reached 0.46 MPa, which was 53% higher than that of the unmodified coatings. In addition, the impermeability of the modified coatings was obviously improved. This was mainly due to the addition of waterborne polyurethane to increase the compactness of the coating.

**Table 2:** The test results of the impermeability of the coatings

Group	Strength (MPa)			Average (MPa)
Blank group	0.28	0.31	0.32	0.30
Modified group	0.45	0.42	0.51	0.46

Table 3 shows the bond strength test results of the coatings. The results indicated that the bond strengths of the modified coating and the unmodified coating with the acid-resistant ceramic tile were 3.08 MPa and 3.84 MPa, respectively. Although the bond strength of the modified coatings was high, the bond strength of the modified coatings with the concrete base was 8% lower than that of the blank group. However, the bond strengths of these coatings were significantly higher than the standard value of 1.2 MPa. Moreover, the bond strength of the modified coating with the acid-resistant ceramic tile was 5.84 MPa, which was 8.5% higher than that of the unmodified coating. This was mainly due to improvement in the strength of the modified coating based on the fact that the bonding surface was not destroyed.

**Table 3:** The bond strength test of the coatings

Group	Material	Bond strength (MPa)
Blank group	Concrete	3.34
Modified group	Concrete	3.08
Blank group	Ceramic tile	5.38
Modified group	Ceramic tile	5.84

### 3.3. Microscopic test analysis

#### 3.3.1. Micro-topography of the coating

**Fig. 2:**
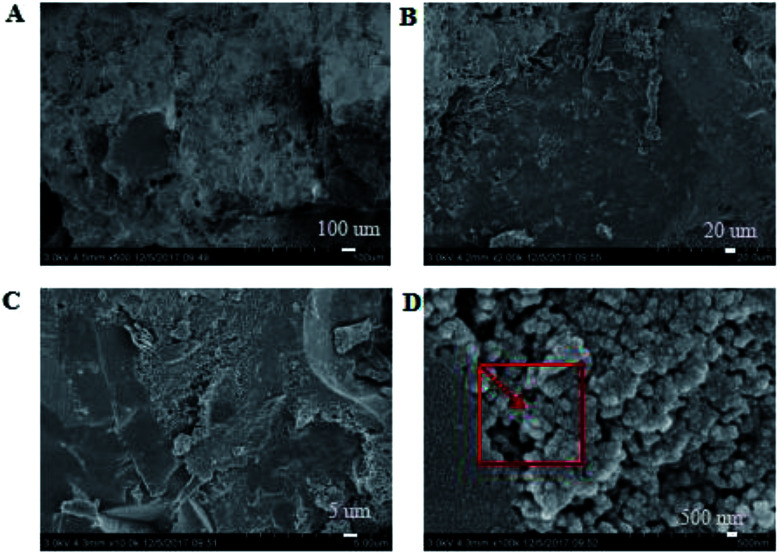
SEM images of the modified coating at normal temperature at the magnifications of ×500 (A), ×2000 (B), ×10 000 (C) and ×100 000 (D).

**Fig. 3:**
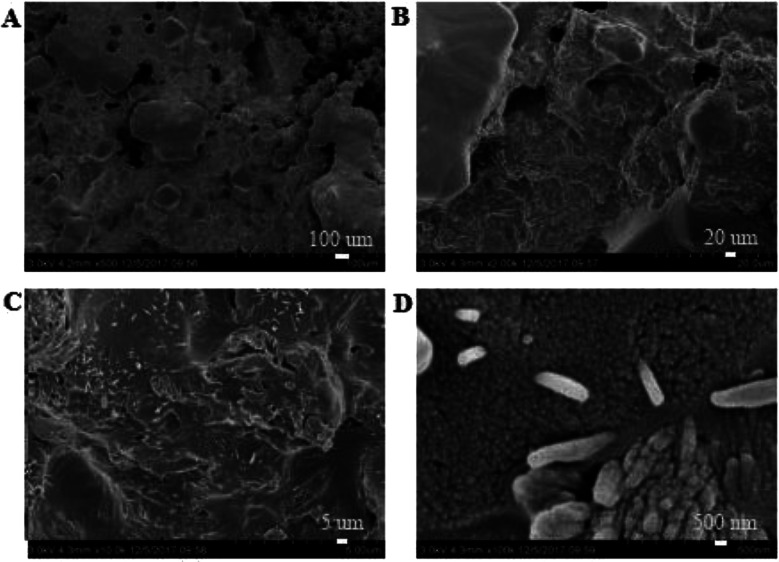
SEM images of the modified coating at resistance temperature at the magnifications of ×500 (A), ×2000 (B), ×10 000 (C) and ×100 000 (D).

SEM images of the coating section obtained at room temperature are shown in Fig. 2 and 5. It can be observed from Fig. 2(C) and (D) that water glass and polyurethane of the modified group coating are well integrated to form an interpenetrating network structure that is tightly wrapped with the filler to form a dense structural system. The void was significantly smaller than that in the case of the blank coating. Therefore, the strength of the coating was improved. This was also in accordance with the strength of the previously modified coating, which was 25% higher than that of the unmodified coating. The water glass adhesive connected the blank group coating. There were a lot of voids in the coating, and the adhesive could not wrap the filler well. Therefore, the coating was not sufficiently dense and strong. SEM images of the coating section obtained at resistance temperature are shown in Fig. 3 and 6. It can be observed from Fig. 3 that polyurethane of the modified group coating does not decompose at the high temperature of 250 °C. The filler still wraps the binder phase tightly, and the high temperature does not destroy the organic–inorganic interpenetrating network. However, it can be observed from Fig. 6 that water in the water glass of the blank group may be further removed due to high temperature, and this may form a large number of irregular honeycomb-like pores, resulting in poor compactness.

**Fig. 4:**
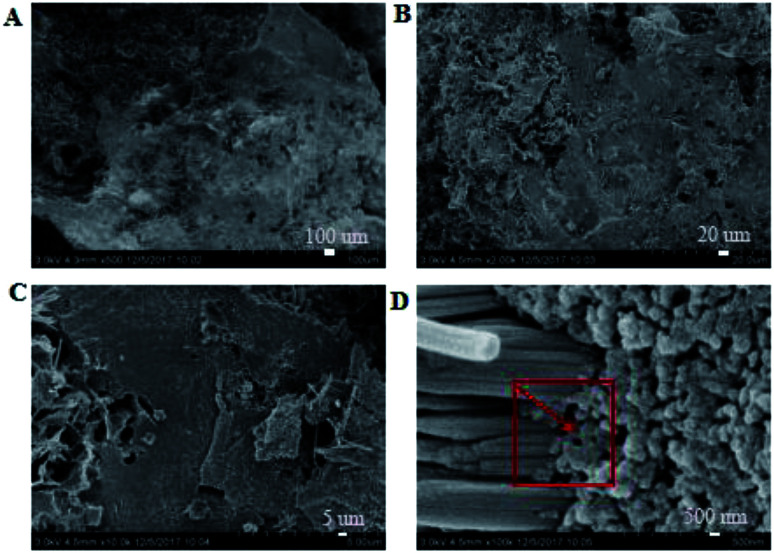
SEM images of the modified coating after acid storage at the magnifications of ×500 (A), ×2000 (B), ×10 000 (C) and ×100 000 (D).

**Fig. 5:**
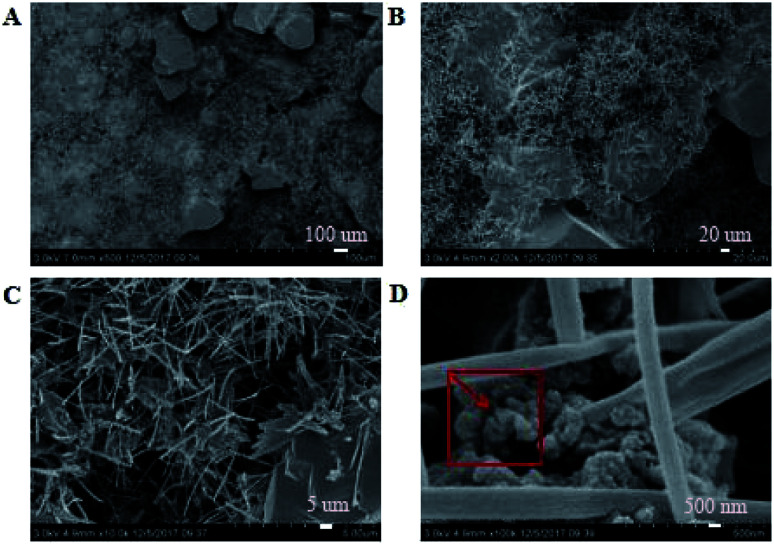
SEM images of the coating belonging to the blank group obtained at normal temperature at the magnifications of ×500 (A), ×2000 (B), ×10 000 (C) and ×100 000 (D).

**Fig. 6:**
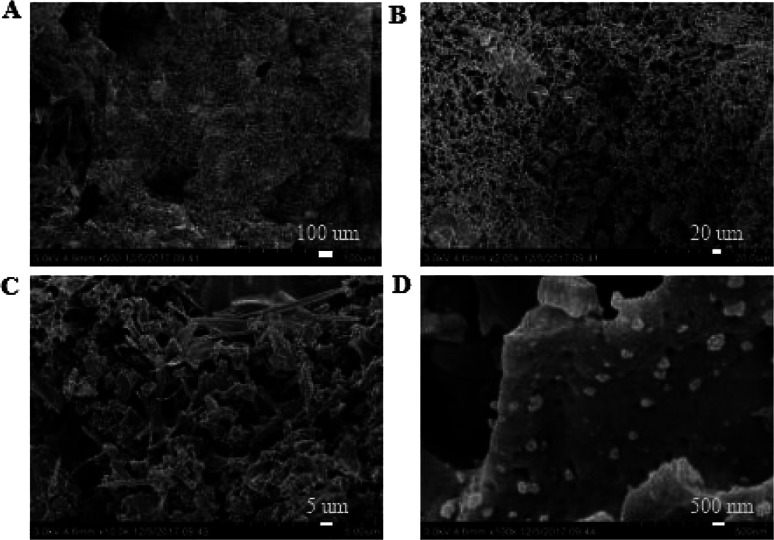
SEM images of the coating belonging to the blank group at resistance temperature at the magnifications of ×500 (A), ×2000 (B), ×10 000 (C) and ×100 000 (D).

**Fig. 7:**
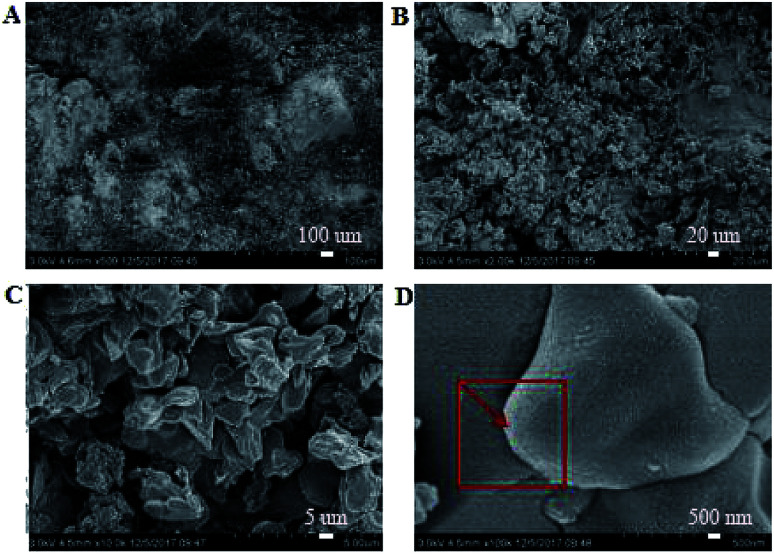
SEM images of the coating belonging to the blank group after acid storage at the magnifications of ×500 (A), ×2000 (B), ×10 000 (C) and ×100 000 (D).

It was observed from Fig. 4 and 7 that after the coatings were immersed in a 50% sulfuric acid solution for 30 days, the interpenetrating network structure formed by the organic–inorganic coating of the modified group coating, as shown in Fig. 4, had no obvious corrosion phenomenon, and only a small number of small voids generated by corrosion existed. The overall structure was still dense. However, the unmodified group coating, as shown in Fig. 7, was obviously dissolved, some structures were corroded, and the gap was further enlarged and deepened. The residual structure was sandy with only a small amount of adhesives to maintain the strength of the structure, and the contact area was small.

#### 3.3.2. Composition analysis

**Fig. 8:**
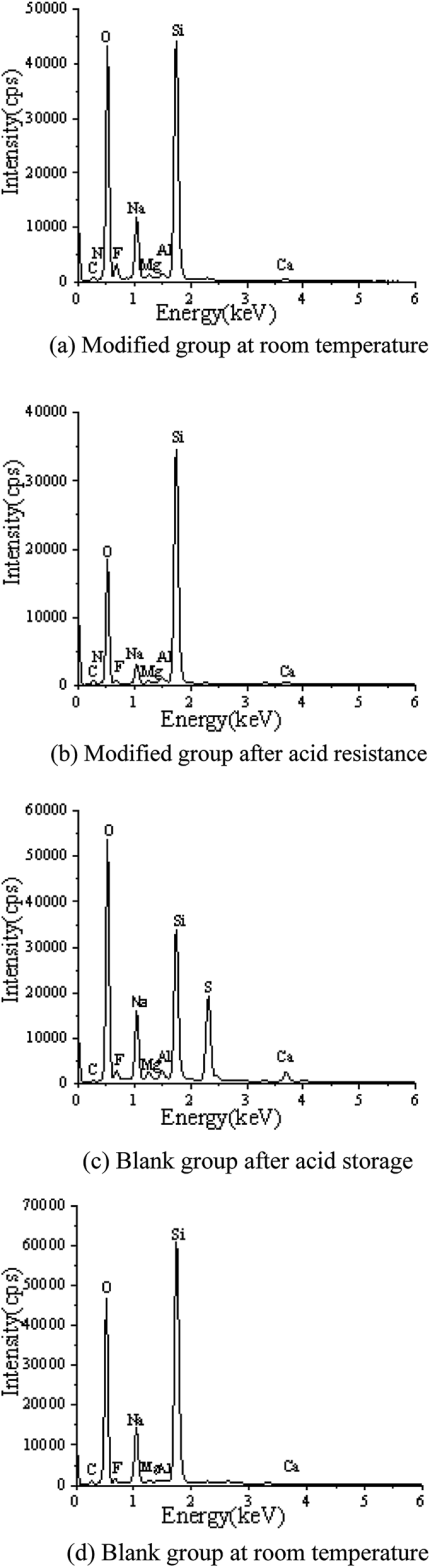
Energy spectra of the coatings at normal temperature and after the acid resistance test.

EDS tests were carried out in the red area marked in the (d) chart in Fig. 2, 4, 5 and 7. It could be observed from Fig. 8 and Table 3 that after storage in sulfuric acid at a 50% concentration for 30 days, no sulfur atom was detected in the modified group coating. However, the sulfur atom percentage of the unmodified group coating was 6.73%. Moreover, the coating sample was 10 mm away from the surface of the test piece; this indicated that the corrosive substance had not intruded into the interior of the modified group but had intruded into the blank group coating. In Table 3, it is shown that the total mass percentage of silicon and oxygen in the modified group increases from 76.28% to 79.26%. However, the total mass percentage of silicon in the blank group decreases from 81.39% to 72.13%. Moreover, it was shown that the modified group obviously resisted corrosion, whereas the unmodified coating was subjected to more severe corrosion in the corrosive solution, resulting in a decrease in the quality of the main bonding element silicon oxide. This was in line with the findings of previous immersion acid strength test, whereby the blank group coating strength decreased significantly and the modified group coating strength remained stable.

### 3.4. Pore structure analysis

**Fig. 9:**
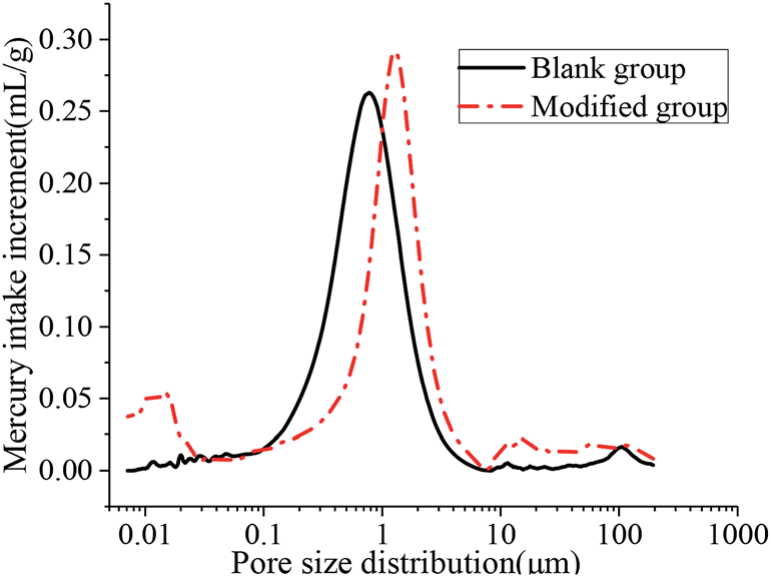
Aperture differential distribution curve.

**Fig. 10:**
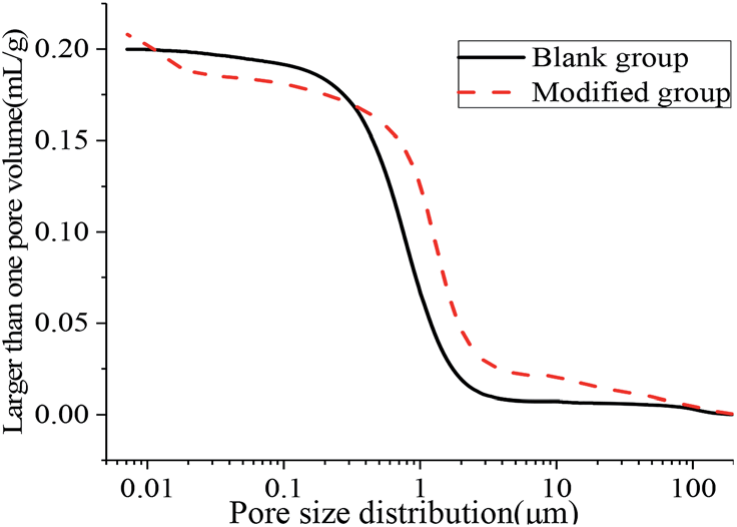
Cumulative pore size distribution curve.

The aperture differential distribution curve and the pore cumulative distribution curve are shown in Fig. 9 and 10, respectively. As observed from Fig. 9, the coating pores were mainly provided by the pores with diameters between 0.1 μm and 3 μm. The peak value of the coatings in the modified group was obvious at the pore diameters between 8 nm and 12 nm, which indicated that waterborne polyurethane could refine the macro-porous structure of the modified group coating to a certain extent and increase the number of the medium holes. However, the porosity of the blank group and the modified group was 33.91% and 33.78%, respectively, and was not significantly changed. The porosities of the blank group and the modified group did not change significantly, and the porosities were 33.91% and 33.78% respectively. Due to the addition of polyurethane, micro-bubbles were produced during the preparation of the coating. The pore size between 10 μm and 100 μm for the modified coating was more than that of the blank coating, resulting in no significant change in the final porosity. Therefore, defoaming agents should be added appropriately during the preparation of the coating. Fig. 10 shows that the cumulative pore size distribution curve of the blank group coating does not change significantly when the pore diameter is less than 100 nm. However, the volume of the modified coating changed obviously; this indicated that the addition of polyurethane reduced the pore size and increased the content of the medium holes; this resulted in higher content of pores with sizes less than 100 nm in the modified coating as compared to that in the blank coating.

Table 4 shows that the most suitable pore sizes for the modified group coating and the blank group coating are 7.12 nm and 11.56 nm, respectively, and the pore structure of the coating is refined. The results of the average pore size and median pore size according to volume, surface area and pore number fraction also showed that the pore size of the modified coating was smaller than that of the blank coating. The addition of polyurethane improved the pore structure of the coating and made the pore distribution of the coating more uniform. Therefore, the strength of the coating was improved. This is consistent with the 25.10% increase in the compressive strength of the modified coatings compared with that of the unmodified coatings.

**Table 4:** Pore structure characteristic values of the coatings

Group	Computing method	Average aperture (nm)	Optimum aperture (nm)	Median aperture (nm)
Blank group	Volume	342	19.95	749.5
	Surface area	342	11.56	158
	Number of holes	342	11.56	16.86
Modified group	Volume	98.19	7.12	1212
	Surface area	98.19	7.12	12.08
	Number of holes	98.19	7.12	10.04

### 3.5. Thermal analysis

**Fig. 11:**
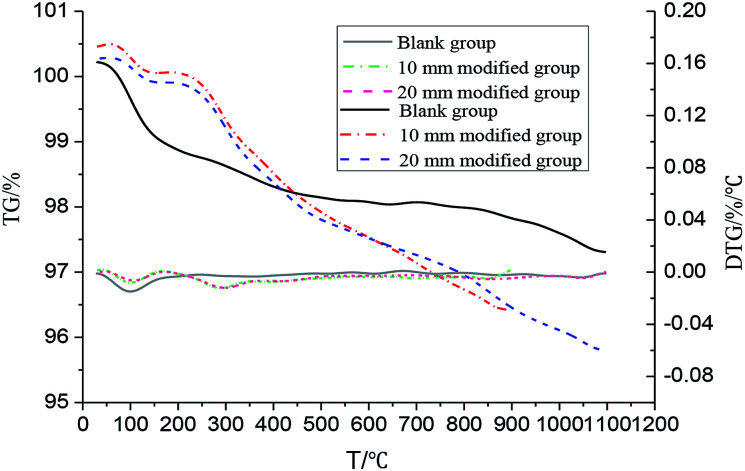
The TG and DTG curves of the coating.

The TG and DTG curves of the coatings are shown in Fig. 11. As observed from Fig. 11, the coating had an obvious weight loss at 70–140 °C. This may be due to the evaporation of trace amount of free water from the sample and the condensation reaction of water in the water glass when the residual portion of the silicic acid gel is increased during curing; in addition, the moisture is further removed, resulting in an obvious weight loss step. However, the weight loss of the modified coating was less obvious than that of the blank coating; this might be because the addition of polyurethane to a certain extent limited the removal of water. The coating of the blank group tended to be stable, and no obvious weight loss phenomenon was found; this was because the coating of the blank group was composed of a filler with excellent temperature resistance and an inorganic adhesive. However, the weight loss of the modified coating at different depths was obvious in the range of 260–380 °C; this was mainly due to the decomposition of polyurethane in the coating. The weight loss curves of the two groups almost coincided; this indicated that polyurethane was evenly distributed in the coating. The weight loss of the modified coating occurred slightly below 250 °C, and the performance of the coating was stable. The results indicated that the modified coating could fully meet the requirement of temperature resistance at 250 °C.

## 4. Conclusions

Herein, the mechanical properties, temperature resistance and acid resistance properties of the modified coating were tested and compared with those of the unmodified coating. The bending and compressive strengths of the coating modified by waterborne polyurethane were improved. Moreover, the flexibility of the coating was greatly improved. The impermeability of the modified coating was 53% higher than that of the unmodified coating. The bond strength of the modified coatings with a concrete and an acid-resistant ceramic tile was significantly higher than the standard value of 1.2 MPa. The SEM images showed that a dense micro-structure with an interpenetrating network was formed. The porosity of the modified coating was obviously smaller than that of the unmodified coating. The EDS analysis showed that the corrosive substance had not intruded into the interior of the modified group but had intruded into the blank group coating. The MIP test showed that the porosity of the modified coating was decreased, and the pore distribution was improved. The TGA analysis showed that the weight loss of the modified coating was small below 250 °C, and polyurethane could meet the requirement of temperature resistance at 250 °C. The waterborne polyurethane-modified silicate-based coating has excellent impermeability and corrosion resistance.

## Conflicts of interest

There are no conflicts to declare.

## Supplementary Material
